# Effectiveness of Novel Oral Anticoagulants Versus Warfarin in Patients With Atrial Fibrillation: A Systematic Review and Meta-Analysis

**DOI:** 10.7759/cureus.61374

**Published:** 2024-05-30

**Authors:** Priyansh Patel, Saloni H Patel, Akshaya Siddegowda, Bhuvana Rasagna Potini, Varsha Miriyala, Diya Patel, Navpreet Singh

**Affiliations:** 1 Department of Cardiovascular Medicine, University of Miami Miller School of Medicine, Miami, USA; 2 Department of Internal Medicine, Smt. Nathiba Hargovandas Lakhmichand Municipal Medical College, Ahmedabad, IND; 3 Department of Internal Medicine, Jagadguru Sri Shivarathreeshwara Medical College, Mysore, IND; 4 Department of Internal Medicine, Kamineni Institute of Medical Sciences, Nalgonda, IND; 5 Department of Internal Medicine, Sri Venkateswara Institute of Medical Sciences, Tirupati, IND; 6 Department of Internal Medicine, Gujarat Medical Education and Research Society, Sola, Ahmedabad, IND; 7 Department of Internal Medicine, Gian Sagar Medical College and Hospital, Patiala, IND

**Keywords:** apixaban, dabigatran etexilate, edoxaban, novel oral anticoagulants, rivaroxaban, warfarin therapy

## Abstract

Atrial fibrillation (AF) is a common cardiac arrhythmia associated with an increased risk of stroke and systemic embolism (SE). Anticoagulation therapy, particularly with vitamin K antagonists (VKA) or novel oral anticoagulants (NOACs), is essential for stroke prevention in patients with AF. However, the comparative effectiveness of NOACs and warfarin remains debatable. Of the 34 studies included, 14 studies involving 166,845 patients were included in the meta-analysis and 20 studies were included in the systematic review. Our findings indicate that NOACs were associated with a significantly lesser risk of stroke/SE with a relative risk (RR) of 0.84 and p=0.0005, and all-cause mortality RR=0.88 and p=0.006. There were no significant differences between major bleeding events with an RR of 0.87 and p=0.22, and serious adverse events (SAE) with RR=1.01 and p=0.35, compared to warfarin in patients with AF. Our meta-analysis demonstrates strong evidence for the superiority in reducing stroke/SE and all-cause mortality of NOACs compared to warfarin. However, no significant differences were identified in the bleeding outcomes or SAEs between the two groups.

## Introduction and background

Arrhythmias and conduction disorders, which are both prevalent and challenging to treat, are affected by risk factors such as aging and obesity. Atrial fibrillation (AF) and ventricular arrhythmia are the most common types of arrhythmias [1]. AF is a persistent cardiac rhythm disruption that has a prevalence of less than 1% among individuals under the age of 60 but increases to over 6% among those aged 80 years or above [2]. AF is the primary cause of cardioembolic stroke, which is the most severe form of ischemic stroke and poses a risk of up to five times greater than other causes [3]. Given the significant mortality and morbidity associated with stroke and venous thromboembolism (VTE), it is crucial to employ effective preventive measures like oral anticoagulants such as warfarin and novel oral anticoagulants (NOACs) [3]. Anticoagulants play a critical role in the treatment of cardiovascular conditions. For many years, vitamin K antagonists (VKA) have been the only available orally active anticoagulants. While VKAs have proven effective, they have several limitations that complicate their use [4]. These limitations have spurred the development of NOACs such as apixaban, rivaroxaban, dabigatran, and edoxaban, which are administered in fixed doses and do not require regular monitoring of clotting levels. These NOACs aim to overcome the challenges associated with VKAs [4]. This meta-analysis aimed to compare the effectiveness and safety of NOACs versus warfarin in AF management by synthesizing evidence from randomized controlled trials and observational studies to inform evidence-based decision-making for optimizing patient outcomes in this challenging clinical landscape.

## Review

Methodology

Our meta-analysis was conducted in accordance with preferred reporting guidelines for systematic reviews and meta-analyses (PRISMA) [5].

Search Strategy and Study Selection

Our search for relevant articles was conducted on databases like PubMed, Medline, PubMed Central, Cochrane, Embase, Scopus, and clinicaltrials.gov using keywords such as “noac,” “warfarin,” “rivaroxaban,” “apixaban,” “dabigatran,” “edoxaban,” “atrial fibrillation,” “therapeutic use,” from their inception until March 31, 2024. All identified articles were imported into Zotero and duplicates were removed. The initial screening of titles and abstracts was followed by full-text screening based on the predefined inclusion and exclusion criteria. Additionally, we manually searched the reference lists of all the included studies. Table 1 shows the search strategies and number of papers identified in each database.

**Table 1:** Literature search PMC: PubMed Central; NOAC: novel oral anticoagulants

No.	Database	Search Strategy	Articles Retrieved
1.	PubMed, Medline, PMC	[NOAC AND Warfarin AND atrial fibrillation]	581
2.	PubMed, Medline, PMC	((("Anticoagulants/adverse effects"[Majr] OR "Anticoagulants/therapeutic use"[Majr] OR "Anticoagulants/toxicity"[Majr] )) AND (( "Warfarin/adverse effects"[Majr] OR "Warfarin/therapeutic use"[Majr] OR "Warfarin/toxicity"[Majr] ))) AND (( "Atrial Fibrillation/drug therapy"[Majr] OR "Atrial Fibrillation/prevention and control"[Majr] OR "Atrial Fibrillation/therapy"[Majr] ))	1373
3.	ClinicalTrials.gov	[Atrial Fibrillation AND Warfarin AND NOAC]	37
		[Atrial Fibrillation AND Warfarin AND Apixaban]	85
		[Atrial Fibrillation AND Warfarin AND Dabigatran]	87
		[Atrial Fibrillation AND Warfarin AND Edoxaban]	38
		[Atrial Fibrillation AND Warfarin AND Rivaroxaban]	95
4.	Cochrane Library	[Anticoagulation AND Atrial fibrillation AND Warfarin]	741

Table 2 shows the eligibility criteria for the studies.

**Table 2:** Eligibility criteria ADHD: attention deficit hyperactivity disorder; AFib: atrial fibrillation; NOAC: novel oral anticoagulants; RCT: randomized controlled trials

No.	Inclusion Criteria	Exclusion Criteria
1.	Articles in English language	Articles including subjects other than humans
2.	Articles discussing the comparative use of NOACs and warfarin in patients with diagnosis of AFib (NOACs include apixaban, rivaroxaban, edoxaban, dabigatran, darexaban)	Articles including subjects with age less than 17 and more than 75 years of age
3.	Articles including human subjects	Articles in any language other than English
4.	Articles including subjects from age 17-75 years of age	Articles like case reports, case series, narrative reviews, systematic reviews, and meta-analysis
5.	Articles including clinical trials, observational studies (cohort, case-control, cross-sectional studies)	Articles including conditions other than AFib that require anticoagulation
6.	Articles including patients with a diagnosis of AFib and treated with either NOAC, warfarin, or anticoagulation	Articles including patients with a diagnosis of AFib and not treated with anticoagulation
7.	Articles with free full-text versions available	Articles including any perioperative use of anticoagulants
8.	Articles were included from all times.	Articles discussing concomitant use of antiplatelet drugs with NOACs and warfarin
9.	Included RCTs with results posted	Articles discussing concomitant administration of heparin and warfarin
10.	-	Articles discussing either only the effects of warfarin or only NOACs
11.	-	Articles discussing the treatment of major bleeding after NOACs or warfarin
12.	-	RCTs with no results posted were excluded
13.	-	Articles discussing the diagnosis of AFib along with psychiatric disorders like depression, anxiety, ADHD, Bipolar disorder, schizophrenia, and mania.

Data Extraction and Outcome Measures

Data from the studies included in the review were obtained using a predesigned Microsoft Excel spreadsheet. One author extracted the data, while the second author cross-checked and entered it into RevMan software for analysis. The extracted data included the author's name, year of publication, study design, sample size, mean age, incidence of stroke, major bleeding, myocardial infarction, deep venous thrombosis, and pulmonary embolism, as well as all-cause mortality and reported serious adverse events (SAE). The primary efficacy outcome of this meta-analysis was the occurrence of stroke/systemic embolism (SE), whereas the occurrence of any major bleeding was the primary safety outcome. All-cause mortality and SAEs were assessed as the secondary outcomes. Any discrepancies in the data extraction process were resolved through discussions between the authors.

Statistical Analysis

We used RevMan Version 5.4.1 (The Cochrane Collaboration, London, United Kingdom) to perform the data analysis. For categorical or dichotomous outcomes, we used the Mantel-Haenszel test for statistical analysis and calculated the risk ratio (RR) as an effect measure with a 95% CI. A significance level of p < 0.05 was adopted. To evaluate the variation in treatment effects across the studies, we utilized both Cochrane Q and I-squared statistics. A p-value of less than 0.10 suggested the presence of heterogeneity, while an I-squared value greater than 50% indicated a significant level of heterogeneity. In cases of significant heterogeneity, we used a random-effects model, whereas a fixed-effects model was used when there was no significant heterogeneity.

Quality Appraisal of the Studies

**Figure 1:**
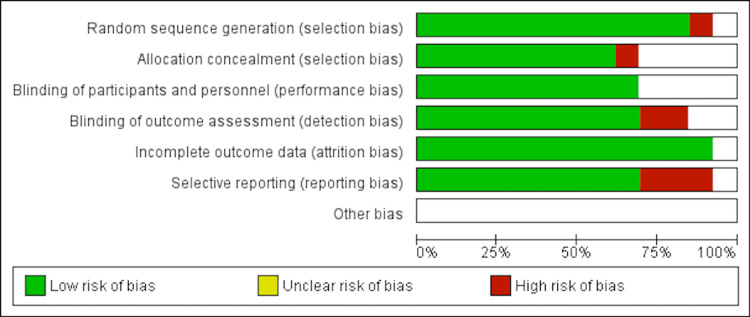
Graphical representation of risk of bias of randomized controlled trials This figure was drawn by the authors themselves using RevMan 5.4.1.

**Figure 2:**
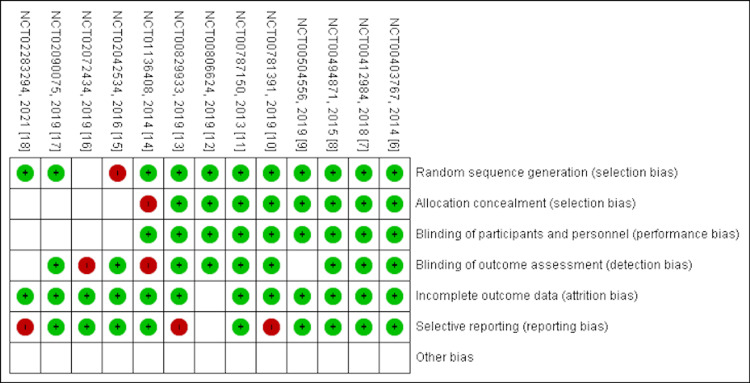
Quality assessment of randomized controlled trials using the risk of bias (ROB-2) tool This figure was drawn by the authors themselves using RevMan 5.4.1.

The shortlisted articles were thoroughly evaluated for quality using established quality appraisal instruments. One author conducted quality checks, while another performed crosschecks. In the event of any disagreements, all co-authors were involved in discussing the issues, and the final decision to accept or reject the paper was made through mutual consensus. Randomized controlled trials [6-18] were assessed using the Cochrane bias assessment tool. Cohort studies [19-25] were assessed using the Newcastle-Ottawa questionnaire. Only studies that satisfied the quality appraisal criteria were included in the meta-analysis and systematic review. Figures 1-2 show the quality appraisal of randomized clinical trials (RCTs) using the risk of bias (ROB) tool.

Table 3 shows the quality assessment of the observational studies using the Newcastle-Ottawa Scale.

**Table 3:** Quality assessment of observational studies using Newcastle-Ottawa tool In the Newcastle-Ottawa tool a star is given to the study for each criterion met. Good quality: 3 or 4 stars in the selection domain and 1 or 2 stars in the comparability domain and 2 or 3 stars in the outcome domain. Fair quality: 2 stars in the selection domain and 1 or 2 stars in the comparability domain and 2 or 3 stars in the outcome domain Poor quality: 0 or 1 star in the selection domain or 0 stars in the comparability domain or 0 or 1 stars in the outcome domain

Name	Study Identifier	Selection	Comparability	Outcome	Overall (Stars)
Pfizer, 2023 [19]	NCT04681482	*	**	**	Poor (5)
Boehringer Ingelheim, 2019 [20]	NCT02081807	***	*	***	Good (7)
Pfizer, 2022 [21]	NCT04435769	****	**	***	Good (9)
Boehringer Ingelheim, 2019 [22]	NCT03254134	**	*	*	Poor (4)
Boehringer Ingelheim, 2017 [23]	NCT02061748	**	*	**	Fair (5)
Boehringer Ingelheim, 2015 [24]	NCT02043808	***	*	***	Good (7)
Boehringer Ingelheim, 2014 [25]	NCT01847547	***	*	***	Good (7)

Results

**Figure 3:**
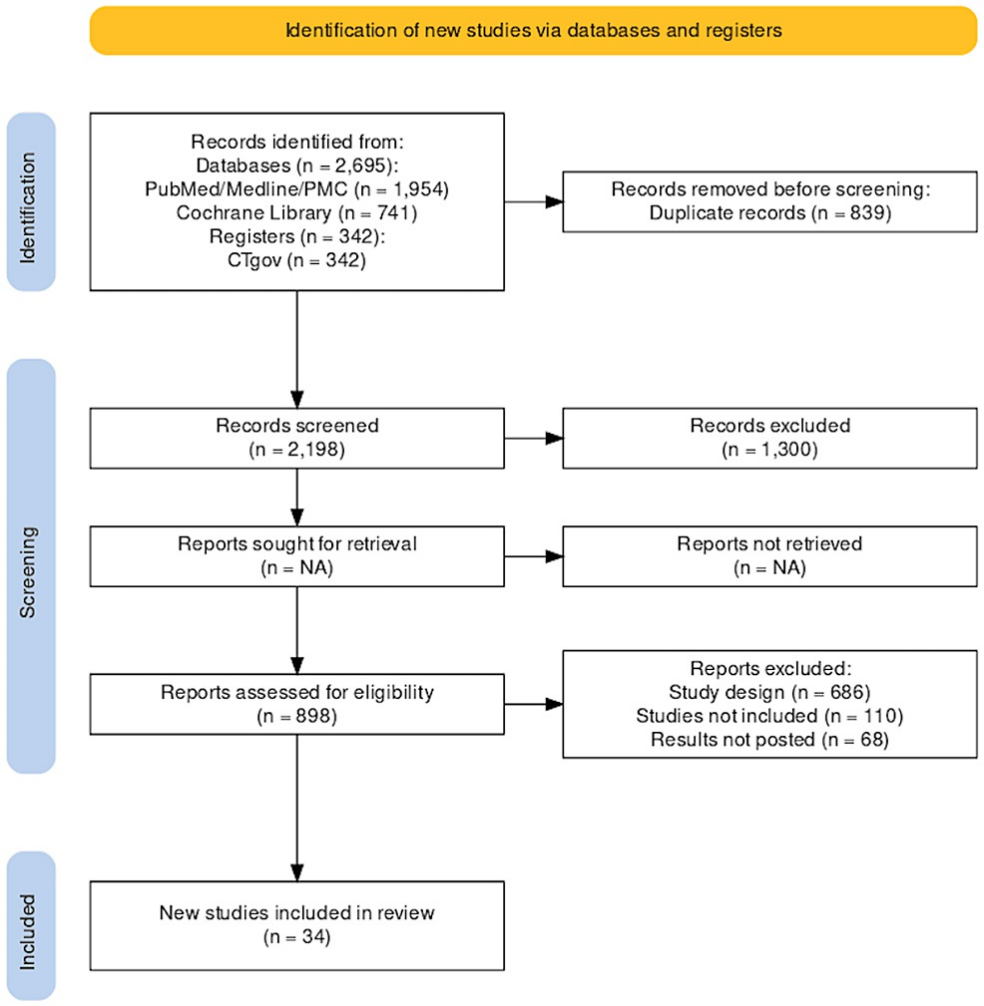
PRISMA flowchart This figure was drawn by the authors themselves.

Through a systematic database search, 3037 studies were identified. After removing duplicates and the studies whose full text was not available, the titles and abstracts of 898 articles were screened. After applying the eligibility criteria and quality assessment, 34 studies were included in this review. Out of 34 studies, 14 were used for meta-analysis with a pooled sample size of 166,845 patients with AF (48,138 in the apixaban group, 12,148 in the dabigatran group, 29,275 in the rivaroxaban group, 16,539 in the edoxaban group, and 60,745 in the warfarin group). The PRISMA flowchart of the included studies is shown in Figure 3 [26].

Individual analyses of eligible studies are presented in Table 4.

**Table 4:** Characteristics of individual studies CAC score: coronary artery calcium score; DVT: deep vein thrombosis; INR: international normalizing ratio; MI: myocardial infarction; NOAC: novel oral anticoagulant; PE: pulmonary embolism; RCT: randomized controlled trial; SAE: serious adverse events; SE: systemic embolism; n1: number of participants in the intervention group; n2: number of participants in the control group

Parent Study Identifier	Study Design	Participants	Mean Age	Males (in %)	Interventions (n1)	Control (n2)	Comments
NCT02081807 [20]	Cohort	221,228	69.2	62	Apixaban (28841) Dabigatran (35124) Rivaroxaban (55059)	Warfarin (102204)	NOACs were associated with lesser events for outcomes like stroke, SE, major bleeding, MI, DVT, and PE.
NCT04681482 [19]	Cohort	107,383	NA	98.6	Apixaban (38756) Dabigatran (12148) Rivaroxaban (21426)	Warfarin (35051)	Apixaban had lesser events for stroke, SE, and major bleeding. Occurrence of SAE was higher in the apixaban group.
NCT00412984 [7,27-30]	RCT	18,201	69.1	64.7	Apixaban (9120)	Warfarin (9081)	Apixaban had lesser events for stroke, SE, major bleeding, and secondary outcomes like MI. Even though all-cause mortality was higher with warfarin, the occurrence of SAE was higher with apixaban.
NCT00787150 [11]	RCT	218	70.3	82.9	Apixaban (148)	Warfarin (74)	Apixaban had lesser events for stroke, SE, and major bleeding. The occurrence of SAE was higher in the apixaban group.
NCT02090075 [17]	RCT	66	57	63.6	Apixaban (33)	Warfarin (33)	Warfarin was significantly better than apixaban for the CAC score.
NCT02283294 [18]	RCT	88	73.5	44.3	Apixaban (41)	Warfarin (47)	All-cause mortality as well as the SAE were higher with warfarin.
NCT04435769 [21]	Cohort	103	79	50.5	Apixaban (40)	Warfarin (63)	Major bleeding events, all-cause mortality, and SAE were seen more with warfarin than with apixaban. Although the cost of outpatient visits was less in the apixaban group than in the warfarin group, the cost of treatment was significantly much higher in the apixaban group. On the other hand, the cost of INR monitoring and cost of admissions to hospital in the first six months were significantly higher in the warfarin group than the apixaban group.
NCT03254134 [22]	Cohort	18,261	76	61.7	Dabigatran (5146)	Warfarin (13115)	Dabigatran was associated with lesser events of stroke, SE, and major bleeding.
NCT01136408 [14]	RCT	147	68.4	88	Dabigatran (90)	Warfarin (57)	Dabigatran was associated with lesser events of stroke, SE, and major bleeding but it was non-inferior to warfarin for secondary outcomes like MI, and all-cause mortality. SAEs were reported more in the dabigatran group than in warfarin group.
NCT02061748 [23]	Cohort	38,499	74.5	55.12	Dabigatran (7646)	Warfarin (30853)	Dabigatran was associated with lesser events of stroke, SE, major bleeding, MI, DVT, and PE. All-cause mortality was found higher in warfarin group.
NCT02043808 [24]	Cohort	25,586	73.9	58.8	Dabigatran (12793)	Warfarin (12793)	Dabigatran had lesser events of stroke, SE, major bleeding, MI, DVT, and PE. All-cause mortality was found higher in warfarin group.
NCT01847547 [25]	Cohort	5982	63.6	69.6	Dabigatran (2991)	Warfarin (2991)	Dabigatran was associated with lesser events of stroke, SE, major bleeding, DVT, and PE. While events of MI were less in the warfarin group than dabigatran group. All-cause mortality was found higher in warfarin group.
NCT02072434 [16,31]	RCT	2149	64.2	65.6	Edoxaban (1067)	Warfarin (1082)	Edoxaban was associated with higher SAE than warfarin group.
NCT00829933 [13]	RCT	525	69	82.5	Edoxaban (396)	Warfarin (129)	Edoxaban was associated with higher SAE than warfarin group.
NCT00781391 [10,32-34]	RCT	21026	70.6	61.9	Edoxaban (14024)	Warfarin (7002)	Edoxaban was associated with higher incidence of stroke and SE.
NCT00504556 [9]	RCT	1143	64.9	62.1	Edoxaban (893)	Warfarin (250)	Edoxaban was associated with lesser events of major bleeding than warfarin.
NCT00806624 [12]	RCT	234	65.1	65.4	Edoxaban (159)	Warfarin (75)	Warfarin had higher events of stroke/SE but lesser events of SAE.
NCT00494871 [8,35]	RCT	1278	71 vs 71.1	80.45 vs 80.5	Rivaroxaban (639)	Warfarin (639)	Rivaroxaban was associated with lower events of stroke/SE, but higher events of major bleeding, MI, and vascular death.
NCT02042534 [15]	RCT	195	70.2 vs 70.6	57.9 vs 59.1	Rivaroxaban (101)	Warfarin (94)	Length of hospitalization was significantly higher for warfarin group than rivaroxaban group.
NCT00403767 [6,36,37]	RCT	14,236	71.2 vs 71.2	60.4 vs 60.3	Rivaroxaban (7109)	Warfarin (7125)	Stroke events were seen less in rivaroxaban compared to warfarin. While events of major bleeding were more in rivaroxaban than warfarin. Events of MI and vascular death were more in warfarin group than rivaroxaban.
NCT00262600 [38,39,40]	RCT	18,113	71.5	63.57	Dabigatran (12091)	Warfarin (6022)	Dabigatran was found beneficial for prevention of stroke/SE and major bleeding.

Primary Efficacy Outcome

**Figure 4:**
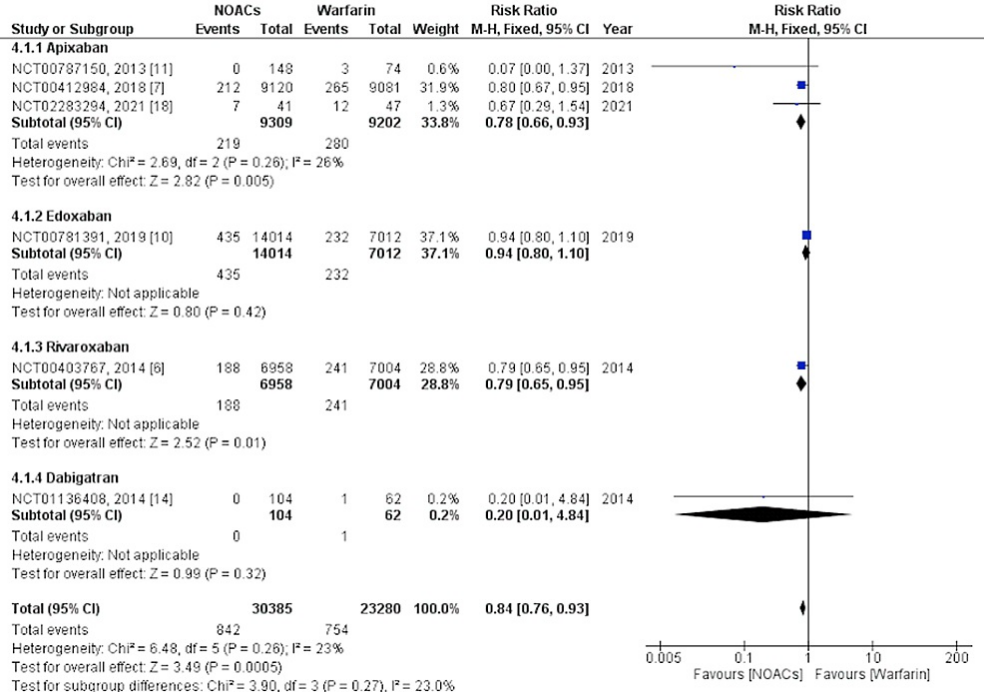
Primary efficacy outcome - Stroke/systemic embolism This figure was drawn by the authors themselves using RevMan 5.4.1.

The primary efficacy outcome was assessed as an event of stroke or SE. The analysis was performed on six studies [6,7,10,11,14,18]. Our findings revealed that the occurrence of stroke/SE was significantly lower with NOACs overall, with an RR of 0.84, 95% CI of 0.76-0.93, I2= 23%, and p=0.0005. We performed further subgroup analysis for individual NOACs compared with warfarin. Subgroup analysis revealed that apixaban (RR= 0.78, 95% CI= 0.66-0.93, I2= 26%, and p=0.005) and rivaroxaban (RR= 0.79, 95% CI= 0.65-0.95, and p=0.01) were significantly associated with lower events of stroke/SE compared to warfarin, whereas edoxaban (RR= 0.94, 95% CI= 0.80-1.10, and p= 0.42) and dabigatran (RR= 0.2, 95% CI= 0.01-4.84, and p=0.32) were not significantly associated with a lower occurrence of stroke/SE. A forest plot showing the subgroup analysis for stroke/SE is shown in Figure 4.

Primary Safety Outcome

**Figure 5:**
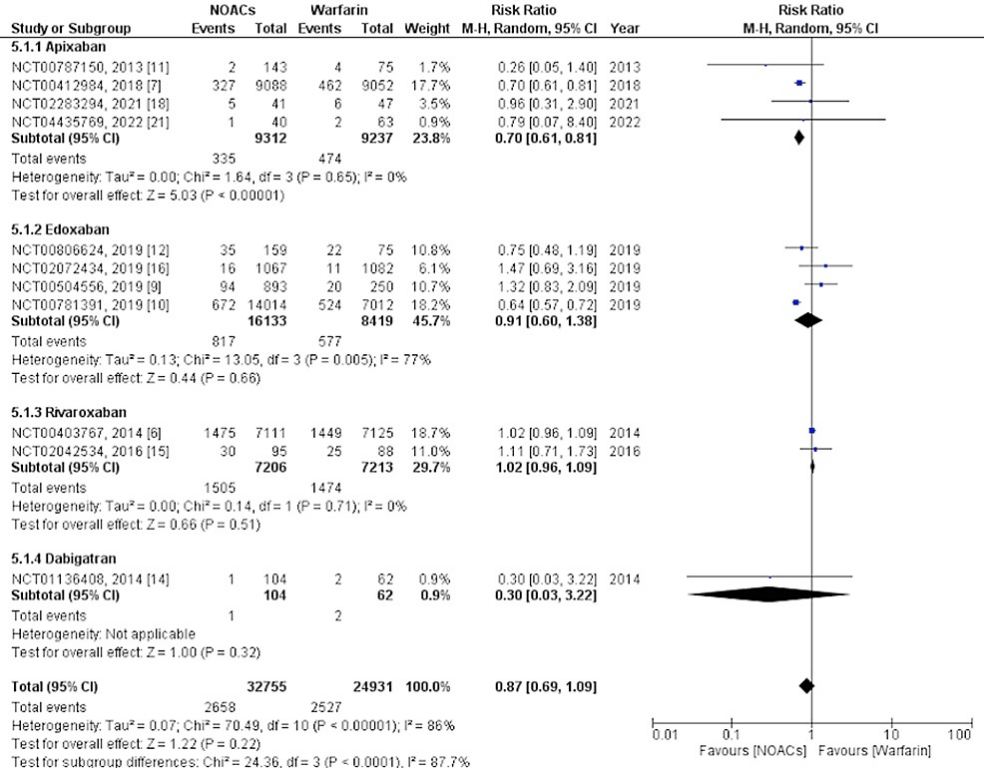
Primary safety outcome - Major bleeding This figure was drawn by the authors themselves using RevMan 5.4.1.

The primary safety outcome was assessed as major bleeding events. Major bleeding was defined as any gastrointestinal (GI) bleeding, intracranial hemorrhage (ICH), fatal or life-threatening bleeding, or any clinically significant nonfatal bleeding. The analysis was performed on 11 studies [6,7,9-12,14-16,18,21]. Our findings revealed that major bleeding was non-significantly associated with lower events with NOACs overall with an RR of 0.87 and a 95% CI of 0.69-1.09, I2= 86%, and p=0.22. We performed further subgroup analysis for individual NOACs compared with warfarin. The subgroup analysis revealed that only apixaban (RR= 0.70, 95% CI= 0.61-0.81, I2= 0%, and p<0.00001) was significantly associated with fewer major bleeding events than warfarin, whereas edoxaban (RR= 0.91, 95% CI= 0.60-1.38, I2= 77%, and p= 0.66), rivaroxaban (RR= 1.02, 95% CI= 0.96-1.09, I2= 0%, and p=0.51), and dabigatran (RR= 0.30, 95% CI= 0.03-3.22, and p=0.32) were not significantly associated with a lower occurrence of major bleeding. A forest plot showing the subgroup analysis for major bleeding events is shown in Figure 5.

Secondary Outcomes

**Figure 6:**
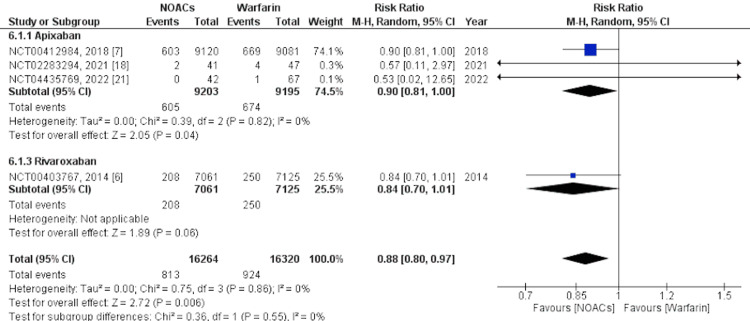
Secondary outcome - All-cause mortality This figure was drawn by the authors themselves using RevMan 5.4.1.

**Figure 7:**
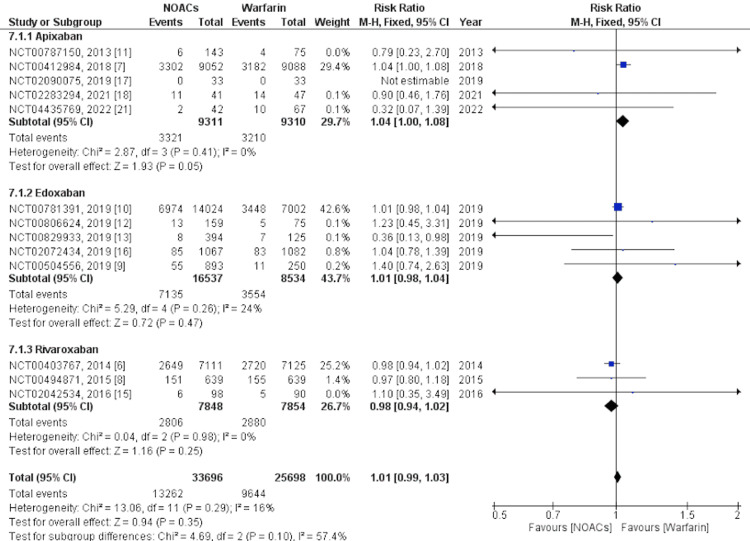
Secondary outcome - Serious adverse events This figure was drawn by the authors themselves using RevMan 5.4.1.

Mortality and SAEs were analyzed as secondary outcomes. Mortality was defined as death from any cause after the first administration of the drug until the end of the follow-up period. SAEs were defined as any reported event that was fatal, life-threatening, or nonfatal but clinically relevant after the first administration of the drug until the end of the follow-up period. For mortality, we analyzed five studies [6,7,14,18,21] and for SAEs, we analyzed 14 studies [6-18,21]. Overall, NOACs were associated with significantly lower mortality than warfarin, whereas SAEs were not significantly associated with a higher occurrence of NOACs. In the subgroup analysis, apixaban was significantly associated with lower mortality, while rivaroxaban was not. Administration of apixaban was significantly associated with higher SAEs. Edoxaban was not significantly associated with higher SAEs while rivaroxaban was not significantly associated with lower SAEs. Forest plots showing the subgroup analysis for mortality and SAEs are shown in Figure 6 and Figure 7, respectively.

Discussion

Overview of Atrial Fibrillation

AF is a prevalent cardiac arrhythmia marked by irregular and often rapid heart rhythms that is significantly associated with age, doubling in successive decades beyond the age of 60 years. With the growing aging population, AF is expected to substantially affect cardiovascular and stroke morbidity and mortality. AF independently increases the risk of stroke four to five times, particularly in those over 80 years of age, contributing significantly to stroke [36]. The prevalence of AF is expected to reach approximately 30% in individuals aged 80 years and above [36]. This condition is intricately linked to adverse outcomes, especially an increased risk of ischemic stroke. The combination of AF with other cardiovascular conditions such as heart failure further increases the risk of mortality and morbidity, including stroke, VTE, congestive heart failure, and compromised quality of life [35]. Although guidelines recommend anticoagulation in patients with AF and heart failure, adherence to clinical practice remains suboptimal [38]. Anticoagulation, which is crucial for stroke prevention in patients with AF, poses challenges, with major bleeding events associated with oral anticoagulants serving as markers of worse outcomes, particularly in patients with concurrent AF and heart failure [27].

Historical Use of Warfarin in AF

Warfarin has long been considered the gold standard for stroke prevention in AF, demonstrating significant efficacy in reducing stroke incidence. Meta-analyses incorporating multiple clinical trials consistently showcase the benefits of adjusted-dose warfarin, highlighting a 64% reduction in stroke risk compared to controls [2]. Despite its effectiveness, the historical use of warfarin is characterized by challenges, including a variable response, potential drug and food interactions, and the need for regular monitoring to adjust doses owing to its narrow therapeutic window [28]. The risk of bleeding, including ICH, has led to its widespread use. Managing the international normalized ratio (INR), especially in patients with left ventricular hypertrophy (LVH), is difficult, impacting both efficacy and safety [39].

Novel oral anticoagulants: Review of literature

Apixaban

Apixaban, a direct oral factor Xa inhibitor, rapidly absorbs with a 12-hour half-life and 25% renal excretion [28]. Granger et al. reported an acceptable side-effect profile, lower discontinuation rate compared to warfarin, and consistently lower major bleeding rates, emphasizing the drug's tolerability and patient adherence, which are essential aspects in long-term treatment strategies [28]. The magnitude of benefit may be influenced by concomitant drug treatment, suggesting a potential interaction between apixaban and other medications [29]. Among individuals diagnosed with AF and possessing at least one additional risk factor for stroke, the administration of apixaban resulted in a reduction in the risk of stroke/SE by 21%, major bleeding by 31%, and mortality by 11% compared with warfarin. In a numerical context, for every 1000 patients subjected to treatment over a span of 1.8 years, the use of apixaban relative to warfarin prevented strokes in six patients, major bleeding in 15 patients, and death in eight patients [28]. In another study, apixaban consistently showed lower major bleeding rates than warfarin. However, the benefits of apixaban seemed to diminish with an increasing number of concurrent drug treatments [29]. Apixaban exhibited higher rates of lower GI and hemorrhoidal bleeding, whereas warfarin showed increased upper GI bleeding. Patients who discontinued anticoagulant treatment for non-major bleeding had a higher 30-day mortality rate than those who continued. The primary non-major bleeding sites included hematuria, epistaxis, GI bleeding, hematoma, and ecchymosis. Clinically relevant non-major bleeding was independently correlated with a 2.18-fold higher risk of major bleeding and a 1.7-fold higher risk of death. Apixaban demonstrated significantly less non-major bleeding than warfarin and was associated with adverse outcomes, including mortality [27]. In another study, apixaban consistently lowered major bleeding rates compared with warfarin across different age groups and maintained its benefits across varying estimated glomerular filtration rates, even among the elderly [30].

Edoxaban

Edoxaban is a reversible direct factor Xa inhibitor with a linear pharmacokinetic profile, 62% oral bioavailability, and 50% renal clearance [32]. To optimize the therapeutic use of edoxaban, Giugliano et al. recommended dose adjustments for patients with low body weight, moderate-to-severe renal dysfunction, or concurrent use of potent P-glycoprotein inhibitors. The drug shows potential interactions with amiodarone and aspirin, affecting its therapeutic effect. GI bleeding rates vary with edoxaban dosage, emphasizing the need for careful consideration [32]. The availability of factor Xa assays and specific reversal strategies in urgent situations could improve safety. The reduction in mortality attributed to edoxaban is primarily linked to a decrease in fatal ICH, indicating its efficacy in preventing critical bleeding events [10]. A trial on acute VTE found that once-daily edoxaban (60 mg, reduced to 30 mg in some cases) was as effective as warfarin in preventing recurrent events with significantly lower bleeding rates. Both edoxaban regimens were non-inferior to warfarin for preventing stroke/SE, and the high-dose edoxaban regimen was more effective. The net clinical outcomes, including cardiovascular events, all-cause mortality, and bleeding, were significantly lower with both edoxaban regimens than with warfarin [32]. In the ENSURE-AF study, edoxaban demonstrated a lower occurrence of stroke/SE, MI, and cardiovascular mortality than warfarin (<1% vs 1%; OR=0.46). The primary safety endpoint, including major and clinically relevant non-major bleeding, was slightly higher in the edoxaban group (16 patients compared to 11 patients in warfarin; OR 1.48) [31]. In another study, more than half of the reduction in all-cause deaths with edoxaban compared with warfarin was directly linked to a reduction in fatal bleeding events and nonfatal major bleeding events contributing to death within 30 days. Notably, warfarin carries a nearly two-fold higher risk of fatal bleeding than edoxaban does. This is significant because edoxaban, (to other NOACs), reduces the incidence of ICH by approximately 50% relative to warfarin, and ICH is associated with a high fatality rate [33].

Dabigatran

Dabigatran, a direct thrombin inhibitor, exhibits consistent treatment effects at 110 mg and 150 mg, regardless of the presence of heart failure, thus emphasizing its versatility [38]. Its substantial renal excretion warrants periodic reassessment of creatinine clearance [39]. In addition to anticoagulation, inhibition of thrombin by dabigatran may have anti-inflammatory and anti-fibrotic properties. The decision to approve only the 150 mg bid dose by the US FDA underscores the prioritization of stroke prevention over extracranial bleeding [40]. The only adverse event that exhibited a significantly higher occurrence with dabigatran than with warfarin was dyspepsia, as reported by Hori et al. [2]. The RE-LY trial established that dabigatran 150 mg twice daily was superior and 110 mg twice daily was non-inferior to warfarin in preventing stroke/SE. Both doses significantly reduced the incidence of hemorrhagic stroke. The higher dose lowered ischemic stroke and cardiovascular mortality, whereas the lower dose caused less major bleeding. The primary differences between the two doses were observed in their impact on major bleeding and ischemic stroke, with a notable 12% reduction in major extracranial bleeding for dabigatran 110 mg compared to 150 mg [40]. In the RE-LY trial, there was no significant interaction between dabigatran treatment effects and the presence or absence of heart failure in terms of efficacy and safety outcomes. Combining data from multiple trials, NOACs showed a significant reduction in stroke/SE compared to warfarin in patients without heart failure but not in those with heart failure. Patients with heart failure experienced no significant increase in major bleeding and had a lower incidence of total bleeding than non-heart failure patients [38]. In the Japanese subset, dabigatran at 110 mg and 150 mg showed a relative risk of 0.52 and 0.25, respectively, for stroke/SE compared to warfarin. For the total study population, the relative risk reduction for stroke/SE with 110 mg and 150 mg, compared to warfarin, was 10% and 35%, respectively. Both dabigatran doses were non-inferior to warfarin in reducing stroke/SE, with 150 mg showing significantly superior risk reduction. Dose-related major bleeding incidence in the dabigatran group was significantly lower with 110 mg than with warfarin in the overall study but not as evident in the limited events in the Japanese population. ICH rates were very low in each group in the Japanese cohort. In summary, the efficacy and safety profiles of dabigatran in Japanese patients with AF at high stroke risk align with the overall study population [2]. In patients without LVH, the primary outcomes of stroke/SE had annual rates of 1.59% with warfarin, 1.60% with dabigatran 110 mg, and 1.08% with dabigatran 150 mg. In patients with LVH, the rates were 3.21% with warfarin, 1.69% with dabigatran 110 mg, and 1.55% with dabigatran 150 mg. The primary outcome was twice as frequent in patients with LVH on warfarin than in those without. Consequently, the lower dose of dabigatran outperformed warfarin in reducing the primary outcome in patients with LVH, whereas the higher dose of dabigatran remained superior to warfarin, regardless of LVH [39].

Rivaroxaban

Rivaroxaban is a direct factor Xa inhibitor with more consistent and predictable anticoagulation effects than warfarin [35]. Bleeding patterns indicate lower rates of critical bleeding events but increased bleeding from GI sites. The clinical impact of rivaroxaban extends beyond anticoagulant efficacy, introducing nuanced considerations in the overall risk-benefit assessment for AF management [36]. In a blinded rivaroxaban regimen compared to an open-label VKA regimen, there were 22 occurrences of stroke/SE events at a rate of 6.42% per year. In contrast, a blinded warfarin regimen transitioning to open-label VKA yielded six events at a rate of 1.73% per year [34]. Despite a higher overall bleeding rate in patients using rivaroxaban, this group experienced fewer critical bleeding events, such as fatal bleeding or ICH, than the warfarin group. Elderly patients with preserved renal function on rivaroxaban showed a slightly more favorable trend in the primary efficacy endpoint than those on warfarin. The differences may be attributed to the intensity of warfarin therapy, following the Japanese guideline recommending a reduced target INR of 1.6-2.6 for individuals aged ≥70 years, potentially contributing to a lower bleeding rate with warfarin compared to rivaroxaban [35]. In patients with AF, rivaroxaban was not inferior to warfarin in preventing stroke/SE. Although there was no significant difference in the major bleeding risk between the two groups, the rivaroxaban cohort experienced fewer occurrences of ICH and fatal bleeding. In contrast, rivaroxaban users had a higher frequency of GI bleeding at the upper, lower, and rectal sites [36]. Patients with a history of MI had worse outcomes in both major and non-major clinically relevant bleeding when treated with rivaroxaban than with warfarin. This was likely associated with the higher prevalence of aspirin therapy in this subgroup. However, a consistent reduction in the occurrence of ICH and fatal bleeding was observed in patients receiving rivaroxaban compared to those receiving warfarin, regardless of their history of prior MI [37].

## Conclusions

This review and analysis provide good evidence for superiority in the efficacy and all-cause mortality of NOACs over warfarin, but we were unable to find any significant difference in the primary safety outcome and serious adverse effects between the two groups. Only apixaban and rivaroxaban were associated with a lesser risk of stroke/SE. Only apixaban was associated with fewer major bleeding events than warfarin. Only apixaban was associated with lower mortality and higher SAEs. No significant difference between NOACs and warfarin for major bleeding events. These findings have important implications for clinical practice and guideline recommendations, suggesting that NOACs should be considered a first-line therapy for anticoagulation in patients with AF. However, further research is warranted to explore the long-term outcomes and comparative effectiveness of the different NOACs in this patient population.
