# Correction: Effectiveness of Novel Oral Anticoagulants Versus Warfarin in Patients With Atrial Fibrillation: A Systematic Review and Meta-Analysis

**DOI:** 10.7759/cureus.c332

**Published:** 2025-10-01

**Authors:** Priyansh Patel, Saloni H Patel, Akshaya Siddegowda, Bhuvana Rasagna Potini, Varsha Miriyala, Diya Patel, Navpreet Singh

**Affiliations:** 1 Department of Cardiovascular Medicine, University of Miami Miller School of Medicine, Miami, USA; 2 Department of Internal Medicine, Smt. Nathiba Hargovandas Lakhmichand Municipal Medical College, Ahmedabad, IND; 3 Department of Internal Medicine, Jagadguru Sri Shivarathreeshwara Medical College, Mysore, IND; 4 Department of Internal Medicine, Kamineni Institute of Medical Sciences, Nalgonda, IND; 5 Department of Internal Medicine, Sri Venkateswara Institute of Medical Sciences, Tirupati, IND; 6 Department of Internal Medicine, Gujarat Medical Education and Research Society, Sola, Ahmedabad, IND; 7 Department of Internal Medicine, Gian Sagar Medical College and Hospital, Patiala, IND

This article has been corrected to fix the following typo in the first paragraph of the Results section: "[...] 36 studies were included in this review." has been corrected to "[...] 34 studies were included in this review".

